# Infective endocarditis mimicking ANCA-associated vasculitis: does it require immunosuppressive therapy? A case report and literature review

**DOI:** 10.1097/MD.0000000000021358

**Published:** 2020-07-17

**Authors:** Xiao-dong Shi, Wan-yu Li, Xue Shao, Li-mei Qu, Zhen-yu Jiang

**Affiliations:** a Rheumatology; b Hepatology, First Hospital of Jilin University; c Hepatopancreatobiliary Medicine, Second Hospital of Jilin University; d Pathology Department, First Hospital of Jilin University, Changchun, China.

**Keywords:** antineutrophil cytoplasmic antibody-associated vasculitis, antiproteinase 3, C-antineutrophil cytoplasmic autoantibody, infective endocarditis, viridans streptococcus

## Abstract

**Rationale:**

In the course of endocarditis, the development of antineutrophil cytoplasmic antibody (ANCA)-mediated disease introduces the dilemma of determining the best treatment approach for immune conditions, whether immunosuppressant therapy should be added to antibiotic treatment has remained controversial.

**Patient concerns:**

A 33-year-old man presented with progressive fever lasting for 7 months, and swelling, pain, and purpura in the arms and legs. The patient showed multiple autoantibodies including cytoplasmic ANCA, antiproteinase 3, rheumatoid factor, and anti-beta 2 glycoprotein I. Blood culture was positive for viridans streptococcus, and renal biopsy revealed glomerulonephritis and interstitial nephritis.

**Diagnosis:**

Endocarditis caused by viridans streptococci, ANCA-associated vasculitis, and congenital ventricular septal defect.

**Interventions:**

In addition to effective antibiotics, he also received twice intravenous corticosteroids and intravenous immunoglobulin therapy, and a low dose of cyclophosphamide. At last, the patient received congenital ventricular septal defect repair and debridement.

**Outcomes:**

The abnormal clinical manifestations, including renal failure and loss of strength, recovered rapidly with corticosteroid therapy in addition to antibiotic treatment. After 6 months without any medications, he remained asymptomatic and was able to live normally.

**Lessons:**

In this case with endocarditis and ANCA-associated vasculitis, we highlighted the importance of biopsy and immunosuppressive therapy. Histopathologic examination is required for diagnosis and treatment in such case. Identifying patients who have endocarditis and ANCA positivity with vasculitis pathologic features will require corticosteroid/immunosuppressives in addition to the antibiotics therapy.

## 1 Introduction

Antineutrophil cytoplasmic autoantibody (ANCA)-associated systemic vasculitis a group of systemic necrotizing vasculitides, which often involve small vessels and lead to few or no immune deposits in affected organs.^[1]^ ANCAs directed against proteinase-3 (PR3) or myeloperoxidase (MPO) are important diagnostic markers for ANCA-associated vasculitis (AAV).^[2]^ ANCAs can also be present in cases of tumors, infections, and other autoimmune diseases. For example, infective endocarditis (IE) can induce clinical manifestations of systemic vasculitis and positive ANCA tests, and thereby mimic AAV.^[3–15]^ In the course of endocarditis, the development of ANCA-mediated disease introduces the dilemma of determining the best treatment approach for immune conditions, because the addition of an immunosuppressive drug to therapy in these patients may increase the risk of infection-related death. Thus, whether immunosuppressant therapy should be added to antibiotic treatment has remained controversial.

Here, we present a case of IE in which ANCA was detected by antigen-specific enzyme-linked immunosorbent assay. To improve our understanding of this disease, we analyzed the present case along with 15 cases previously reported in the literature. This case study has been approved by the Ethics Committee of The First Hospital of Jilin University. And the patient in the present case provided written consent for inclusion in the study and for publication of this case report.

## 2 Case presentation

A 33-year-old man was hospitalized on October 10, 2018 for progressive fever lasting for 7 months. He presented with a temperature of 38°C to 40°C; swelling and pain in the wrists, knees, and ankles; and the purpura in the skin of the lower extremities. He had a previous medical history of congenital heart disease with a congenital ventricular septal defect (VSD). During examination, grade 3/6 systolic murmurs were heard in the mitral valve area, and skin petechia was noted in the lower limbs.

Blood tests revealed an increase in creatinine level from a baseline of 0.86 mg/dL (76 μmol/L) to 3.92 mg/dL (347 μmol/L) (reference range [RR] 57–97 μmol/L) over 10 days. Blood and protein appeared to be positive from urinalysis, and cytoplasmic ANCA (C-ANCA) 1:10 (RR < 1:10), PR3 level of 176.32 AU/mL (RR, 0–15 AU/mL). The measured anti-beta 2 glycoprotein I (β2GPI) level was 73 RU/mL (RR, 0–20 RU/mL). The antinuclear antibody test results were negative. The erythrocyte sedimentation rate and C-reactive protein level were elevated at 49 mm/h (RR < 10 mm/h) and 72.8 mg/L (RR < 20 mg/L), respectively. Virologic and fungal test results were negative. The complement C3 level was low at 0.67 g/L (RR, 0.9–1.8 g/L). A full blood count showed a hemoglobin level of 80 g/L, a white blood cell count of 7.26 × 10^9^/L, and a platelet count of 62 × 10^9^/L. Multiple blood cultures were positive for viridans streptococcus.

**Figure 1:**
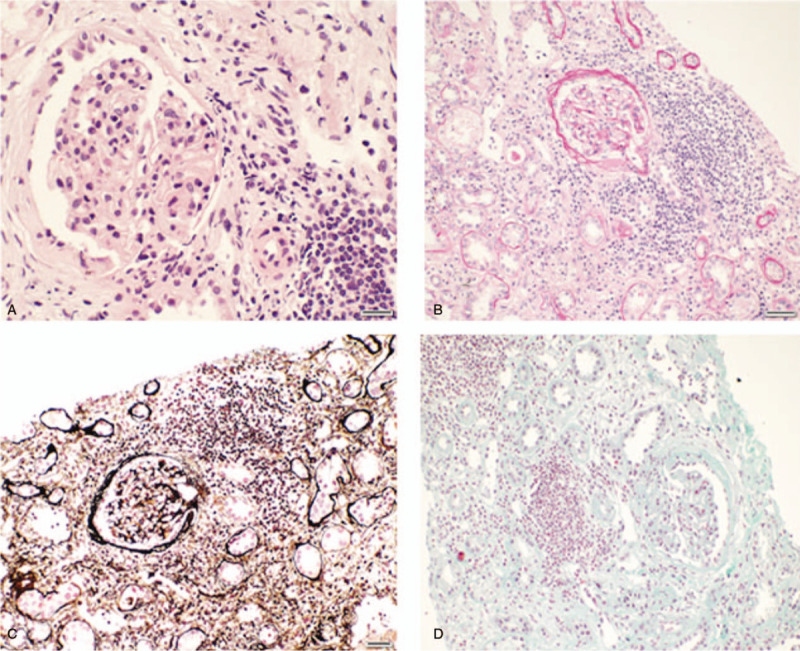
Light microscopic findings on renal biopsy. (A) Hematoxylin and eosin stain (×400). (B) Periodic acid-Schiff stain. (×200). (C) Hexamine silver-PAM stain (×200). (D) Masson trichrome stain (×200).

Splenomegaly was confirmed by ultrasound. Computed tomography of the chest revealed multiple nodules in each lobe of both lungs, about 0.5 to 1.5 cm in size, with a small amount of pleural effusion on both sides. Doppler echocardiography showed no valve vegetations. However, the continuity of the periventricular septal membrane was interrupted, and a 5.7-mm-wide defect had formed, reflecting the patient's congenital heart disease-ventricular septal defect. Kidney biopsy showed necrotizing glomerulonephritis with crescentic glomerulonephritis and interstitial nephritis. Immunofluorescence staining showed the tissue was IgA (+/−), IgG (−), C3 (−), C4 (−), C1q (−), and Flb (−), and electron microscopy showed no deposits (Fig. 1).

The patient was treated initially with antibiotics, followed by moxifloxacin 3 days later, based on the drug sensitivity test result. The patient's fever subsided soon after initiation of antibiotics, but his platelet count, erythrocyte count, and ANCA index did not improve. Furthermore, progressive renal insufficiency developed, and his serum creatinine level increased to 417 μmol/L after 7 days. A subsequent renal biopsy revealed crescentic glomerulonephritis and interstitial nephritis with negative immunofluorescence (IgA (+/−), IgG (−), C3 (−), C4 (−), C1q (−), and Flb (−)), which is consistent with ANCA-associated pauci-immune glomerulonephritis.

The possibility of pursuing aggressive treatments was discussed with the patient's family based on the discovery of the ANCA-mediated disease (glomerulonephritis). In addition to antibiotics, he received intravenous corticosteroids (methylprednisolone, 40 mg/d) and intravenous immunoglobulin therapy. On this regime, his purpura, joint pain, and renal failure reversed rapidly, and his strength and nutrition recovered. Repeated cardiac ultrasound showed no valve vegetations. Subsequently, the corticosteroid dose was rapidly reduced to 30 mg/d given the patient's rapid improvement. And the patient received oral prednisolone first at 20 mg daily for 5 days and then at 10 mg daily for 5 days consecutively.

However, 4 months later, the patient experienced disease relapse, with the return of fever, purpura rash, weakness, and edema of the lower limbs. The patient was rehospitalized. Re-examination of cardiac ultrasound showed several patchy strong echoes at the right ventricular part of the septal defect in the heart, which swayed slightly with the cardiac cycle, indicating the formation of vegetations at the septal defect. Abdominal ultrasonography suggested that the spleen was greatly enlarged. Subsequent investigations revealed that the C-reactive protein level was elevated to 74.7 mg/L. C3 was low at 0.17 g/L, C4 was 0.04 g/L (RR, 0.1–0.4). A full blood count showed a hemoglobin level of 44 g/L, a white blood cell count of 2.4 × 10^9^/L, and a platelet count of 99 × 10^9^/L; as well as a serum creatinine level of 729 μmol/L, an ANCA-PR3 concentration of 62 AU/mL, and a C-ANCA ratio of 1:10. The blood culture was also positive for viridans streptococcus.

The patient was treated with antibiotics and intravenous immunoglobulin again. And after the fever had improved, he received a moderate dose of methylprednisolone (60 mg/d) intravenously for 3 days and a low dose of cyclophosphamide (0.2 g/wk), with gradual dose reduction, along with regular dialysis. One month later, after the short-term treatment with the low-dose glucocorticoid combining with continuous antibiotics, the patient's fever and renal insufficiency dramatically improved. We discontinued the intermittent hemodialysis when his creatinine level had reached 276 μmol/L. Then, he received VSD repair and debridement. After the surgery and a series of maintenance treatments, his renal function gradually recovered, with no presentation of edema. The glucocorticoid therapy was gradually reduced until it was stopped. After approximately 6 months without any medications, he remained asymptomatic and was able to live normally. His repeated echocardiogram at the 6-month follow-up was consistent with his postsurgery ultrasound.

## 3 Literature review

We performed a Medline literature search to identify cases mimicking AAV reported from 1998 to 2017. We found 15 representative cases in 13 papers, refer to summary in Table 1. The 15 cases included 14 men and 1 woman. Three of the patients were young, aged 16 to 24 years, while the other 12 cases were older, aged 47 to 83 years. All of these patients presented with the initial symptoms of fatigue and fever. Purpura/rash was found in 10 (66.6%) patients, and edema of the lower extremities occurred in 7 (46.6%). Splenomegaly and weight loss were noted in 6 (40.0%) cases. Four (26.6%) individuals complained of myalgias/arthralgia, and macrohematuria was observed in 2 (13.3%) patients. One young man developed dry gangrene and ischemia in the forefoot. Nine (60.0%) patients had a previous history of heart disease. And a predisposing factor (dental treatment, etc) was found in 4 patients (26.6%).

**Table 1:**
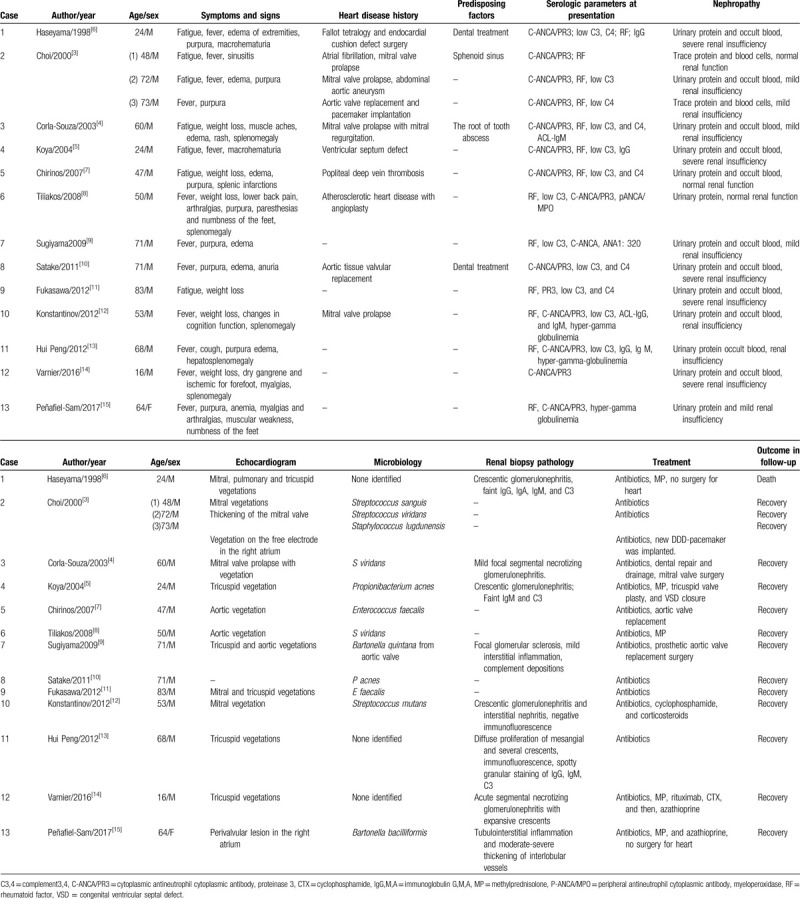
Summary of infective endocarditis patients positive for ANCA.

Laboratory findings in all 15 patients indicated nephropathy. Urinary protein, occult blood, and renal insufficiency were noted in 13 (86.6%) patients. Notably, all 15 patients were C-ANCA/PR3-positive. Elevated RF levels occurred in 13 (86.6%) cases, and a low complement level (C3/C4) was noted in 12 (80.0%) patients. Five (33.3%) patients had hyper-gamma globulinemia. Blood cultures were conducted in all 15 cases, and a causative microorganism was identified in 12 (80.0%) cases, with streptococcus being the leading microorganism. Cardiac valvular vegetation was identified by echocardiogram in 14 patients. Renal biopsy was conducted in 8 cases. Histologic analysis of the specimens showed crescentic glomerulonephritis in 5 cases, and immunofluorescence studies showed faint C3 and IgM expression in 3 cases.

In terms of outcomes, only 1 patient among the 15 cases died of renal failure,^[6]^ while all of the other patients survived and recovered after therapy. Five patients underwent cardiac surgery in addition to receiving antibiotic therapy, and 6 cases that showed significant renal pathology changes also received corticosteroid/immunosuppressive agents.

## 4 Discussion

In this case, a patient with ANCA-mediated disease triggered by infective endocarditis developed a rapid clinical decline during appropriate antibiotic treatment, and the disease was relieved by the addition of corticosteroids and immunosuppressive agents.

Our literature search identified several other cases that also showed rapid improvement in patients with infective endocarditis and ANCA-mediated disease with the addition of immunosuppressive regimes (Table 1).^[5,8,12,14,15]^ As their clinical symptoms worsened, these patients also underwent renal biopsies, which provided histologic evidence of ANCA-mediated disease, including crescentic glomerulonephritis and acute interstitial nephritis (Fig. 1)^[5,12,14,15]^ The kidney is an organ commonly involved in infective endocarditis. Biopsy of the kidney in patients with endocarditis and renal disease can reveal a variety of histologic manifestations, such as postinfectious or vasculitic glomerulonephritis, renal infarction, acute interstitial nephritis, acute tubular necrosis, and others.^[16]^ The histopathologic features of the biopsied kidney influence the choice of treatment. Pauci-immune glomerulonephritis may require corticosteroids and other immunosuppressants in addition to antibiotics. Postinfective glomerulonephritis is usually responsive to antibiotic therapy for endocarditis and, in some cases, also requiring corticosteroids.^[6]^ Our case and some cases previously reported in the literature provide additional evidence for the effectiveness of this treatment. Interstitial nephritis also has been found in patients with ANCA-related disease^[17,18]^ and may require corticosteroids and immunosuppressants in addition to adjustment of antibiotics.

Infective endocarditis can induce the production of a variety of autoantibodies (Table 2), and the clinical manifestations of these patients develop into either chronic infection or vasculitis. In the present case, the patient was positive for a variety of autoantibodies during the course of the disease, including anti-β2GPI, RF, PR3-ANCA, low complement, and others. Many reports have found that ANCA positivity associated with infectious diseases can present as double ANCA positivity (high PR3, low MPO titer), accompanied by antinuclear antibody, anticardiolipin antibody, high globulin, anti-β2GPI antibody, and complement consumption.^[19]^ Endocarditis with similar clinical manifestations to AAV is more common with PR3-ANCA, and other non-organ-specific autoantibodies (such as antinuclear antibodies, etc) may also be present.^[20]^

**Table 2:**
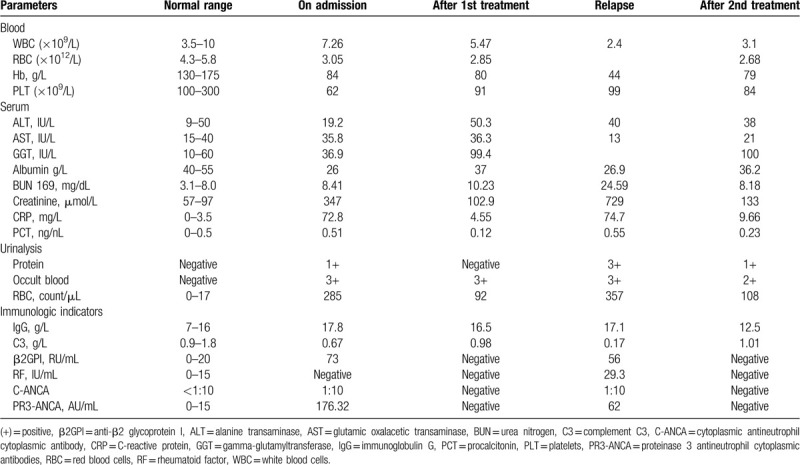
Laboratory values of the patient.

The patient in the present case had a precipitating factor of congenital heart disease, and the pathogen identified by blood culture was viridans streptococcus. Most of the previously reported cases also had a history of heart valve disease (Table 1), with cardiac valvular vegetation found in 14 patients. The pathogens identified in the previous cases were mainly *Staphylococcus* and *Streptococcus*. Among the pathogens known to mediate ANCA production, *Streptococcus* and *Staphylococcus* are dominant,^[3,4,7,8]^ but others include *Tuberculosis bacilli*, *Legionella*, and other invasive aspergillosis, hepatitis C virus, parvovirus B19, and *Bartonella*, which can cause PR3/MPO-ANCA positivity.^[10,20]^ Therefore, timely microbial culture or pathogen-specific antibody detection is important, especially in patients with heart disease.

How do the bacteria of infective endocarditis cause ANCA abnormalities that can lead to vasculitis? Sustained bacterial infection can mediate activation of polyclonal B lymphocytes, which in turn produce autoantibodies, including ANCA, antinuclear antibodies, RFs, and cold globulin. In the context of pathogen infection, vascular damage may activate expression of cytoplasmic enzymes (PR3 and MPO) in endothelial cells and polymorphonuclear giant cells.^[5]^ Therefore, a low titer or nonspecific ANCA can be detected in many patients with infectious diseases. In general, testing for ANCA gradually turns negative as the infection is cured, rarely leading to the generation of sufficient ANCA levels that cause the irreversible histopathologic damage to the characteristics of AAV.^[6]^*Staphylococcus* or *Streptococcus*, as a common pathogenic microorganism of infective endocarditis, may mediate the generation of ANCA via the following mechanisms: the partial genetic sequence of the bacteria complements the important sequence of PR3, which mediates ANCA production through “indirect molecular simulation”^[21–23]^; the unmethylated oligodeoxynucleotides of the bacteria activate B lymphocytes through Toll-like receptor 9, which produce ANCA^[23]^; activated neutrophils release chromatin fibers to form an “extracellular neutrophil trap,” which is an important mechanism for resistance against pathogenic microorganisms^[24]^; and extracellular neutrophils may be released during the initial ANCA-associated immune response and cause vascular damage, which was found to be closely related to high-titer PR3-specific cytoplasmic ANCA.^[25]^ Neutrophil extracellular traps may play a leading role in the development of glomerulonephritis related to bacterial infective endocarditis associated with cytoplasmic ANCA/anti-PR3 positivity. These autoimmune serologic abnormalities are closely associated with pathogenic microorganisms that may contribute to immunodeposition in kidney damage from ANCA-positive infectious diseases.^[26]^

Pathologic examination of the renal biopsy of this patient showed necrotizing glomerulonephritis with crescentic glomerulonephritis and interstitial nephritis, and negative immunofluorescence staining. In addition to antibiotic therapy, we administered timely corticosteroid and immunosuppressant therapy, and the patient's condition improved rapidly. Among the cases identified in our literature search, 6 patients were treated with corticosteroid/immunosuppressants and eventually improved according to significant pathologic changes in the kidney. Thus, immunosuppressants may be required in some patients with infective endocarditis who develop ANCA-mediated renal disease. Histologic identification of the type of renal disease is imperative for selecting the appropriate treatment.

Similar clinical manifestations of vasculitis and ANCA detection are not necessary for clinicians to completely isolate ANCA-positive infectious diseases from AASV. At this point, screening and exclusion of infection factors should be actively conducted to avoid misdiagnosis of AASV due to the presence of ANCA. These infectious diseases may be a stage in the development of infectious factors that eventually lead to AASV: during the course of the disease, with the clearance of the pathogen, the autoantigen tolerance, which was temporarily broken, is restored. Accordingly, the autoantibody levels decrease significantly or disappear, and the patient's condition improves. If these pathogens are not cleared in a timely manner and are able to cause chronic infection, the autoantigen tolerance will be impaired, leading to autoimmune disorders, and the continuous production of ANCA will eventually lead to the pathologic damage typical of AASV.^[27]^

For most bacterial infections, treatment with effective antibiotics and/or surgical treatment can usually prevent the progression of inflammation and suppress the ANCA titers gradually to even undetectable levels. However, there have been numerous reports of ANCA-positive infective endocarditis patients who received immunosuppressant therapy in addition to rational antibiotic therapy.^[5,12,28]^ The use of corticosteroid/immunosuppressants depends more on histologic evidence than on the efficacy of anti-infection therapy.^[28]^ Take endocarditis with ANCA-positive involvement of the kidney as an example, the pathologic types mainly include renal infarction, glomerulonephritis, acute tubular necrosis, and acute interstitial nephritis, etc.^[16]^ Treatment depends on histologic evidence: post-infection glomerulonephritis responds well to antibiotic therapy, but some cases do require corticosteroid therapy. In the treatment of pauci-immune glomerulonephritis or vasculitis, the use of corticosteroid and immunosuppressants in addition to anti-infection therapy is widely accepted. However, standardized antimicrobial and immunosuppressant therapies require further study.

## 5 Conclusion

In the course of infective endocarditis triggered ANCA-mediated manifestations, histopathologic examination is required for diagnosis and is a key basis for treatment in such case. Identifying patients who have endocarditis and ANCA positivity with vasculitis pathologic features will require corticosteroid/immunosuppressives in addition to the antibiotics therapy.

## Author contributions

**Conceptualization:** Xiao-dong Shi, Zhenyu Jiang.

**Data curation:** Xiao-dong Shi, Wan-yu Li, Li-mei Qu.

**Investigation:** Xiao-dong Shi, Wan-yu Li, Xue Shao.

**Methodology:** Xiao-dong Shi, Xue Shao, Li-mei Qu.

**Supervision:** Li-mei Qu, Zhen-yu Jiang.

**Writing – original draft:** Xiao-dong Shi, Wan-yu Li.

**Writing – review & editing:** Li-mei Qu, Zhen-yu Jiang.
